# Evaluation at scale of microbiome-derived metabolites as biomarker of flavan-3-ol intake in epidemiological studies

**DOI:** 10.1038/s41598-018-28333-w

**Published:** 2018-06-29

**Authors:** Javier I. Ottaviani, Redmond Fong, Jennifer Kimball, Jodi L. Ensunsa, Abigail Britten, Debora Lucarelli, Robert Luben, Philip B. Grace, Deborah H. Mawson, Amy Tym, Antonia Wierzbicki, Kay-Tee Khaw, Hagen Schroeter, Gunter G. C. Kuhnle

**Affiliations:** 1 Mars, Inc., McLean, VA USA; 2 Department of Nutrition, UC Davis, CA USA; 3 MRC Epidemiology Unit, University of Cambridge, Cambridge, UK; 4 Department of Public Health and Primary Care, University of Cambridge, Cambridge, UK; 5 LGC, Newmarket Road, Fordham, UK; 6 Department of Food and Nutritional Sciences, University of Reading, Reading, UK

## Abstract

The accurate assessment of dietary intake is crucial to investigate the effect of diet on health. Currently used methods, relying on self-reporting and food composition data, are known to have limitations and might not be suitable to estimate the intake of many bioactive food components. An alternative are nutritional biomarkers, which can allow an unbiased assessment of intake. They require a careful evaluation of their suitability, including: (a) the availability of a precise, accurate and robust analytical method, (b) their specificity (c) a consistent relationship with actual intake. We have evaluated human metabolites of a microbiome-derived flavan-3-ol catabolite, 5-(3′,4′-dihydroxyphenyl)-[gamma]-valerolactone (gVL), as biomarker of flavan-3-ol intake in large epidemiological studies. Flavan-3-ols are widely consumed plant bioactives, which have received considerable interest due to their potential ability to reduce CVD risk. The availability of authentic standards allowed the development of a validated high-throughput method suitable for large-scale studies. In dietary intervention studies, we could show that gVL metabolites are specific for flavan-3-ols present in tea, fruits, wine and cocoa-derived products, with a strong correlation between intake and biomarker (Spearman’s r = 0.90). This biomarker will allow for the first time to estimate flavan-3-ol intake and further investigation of associations between intake and disease risk.

## Introduction

Flavan-3-ols are a group of phenolic compounds that have been under intensive investigation for their effect on vascular function and the potential to reduce the risk of cardio-vascular diseases^1,2^. Numerous small-scale, short-term studies using surrogate markers of health have been conducted, but it is not clear whether these results provide a reliable estimate of the effect on disease risk reduction and primary disease prevention in the general public^3,4^. This can only be achieved with large, long-term prospective observational epidemiological studies and intervention studies such as COSMOS (NCT02422745). The advantage of the former is that they can provide information about flavan-3-ol intake as part of the habitual diet and that they can make use of data or samples already collected. As of now, epidemiological studies on flavanols, which relied on the validity of the assessment of dietary intake and food content data bases^5^, have shown modest or no relationship between flavanol intake and a decreased risk of cardio-vascular diseases^6^. While dietary intake assessments are often based on a combination of dietary self-reporting and food-composition databases, this approach has well-known limitations^7,8^.

An alternative method for the assessment of dietary intake are nutritional biomarkers. They are objective and measurable characteristics of an individual that are indicative of the intake of a given nutrient or dietary constituent^9^. Nutritional biomarkers can be broadly divided into two categories^10–13^: recovery biomarkers, that allow the accurate estimation of actual intake, such as the use of urinary nitrogen for protein intake^14^, and surrogate or concentration biomarkers that allow the ranking of a study population according to their intake in order to improve dietary assessment or investigate associations with disease risk. Most biomarkers currently used in the context of assessing the intake of bioactives, belong to the latter category^11,12^. These nutritional biomarkers are often based on the concentration of specific metabolites of the compounds of interest in plasma, serum or urine, an approach that we have used previously for isoflavones^15,16^.

In recent years, various human flavan-3-ol metabolites have been identified^17^, and the microbial ring-fission catabolite 5-(3′,4′-dihydroxyphenyl)-γ-valerolactone (gVL)^18,19^, in particular its phase II metabolites gVL-3′/4′-sulphate and gVL-3′/4′-*O*-glucuronide (gVLM), have emerged as strong candidates for a nutritional biomarker of flavan-3-ol intake in humans. This is in based on their pharmacokinetic characteristics, as their half-life is sufficient to result in steady-state like conditions with regular consumption^17,20^.

Here, we use criteria proposed by IARC^10^ and the Institute of Medicine (IOM)^21^ to evaluate the suitability of gVLM as a nutritional biomarker of flavan-3-ol intake, in particular that:

i. gVLM can be measured accurately and precisely in the relevant human biospecimen, e. g. plasma or urine;
ii. gVLM are specific for flavan-3-ols;
iii. gVLM concentration or excretion reflect changes in dietary intake consistently and therefore are prognostic of intake

Furthermore, for use in large-scale epidemiological studies, the analytical method should be robust and suitable for high-throughput analysis.

In this study, we use these criteria to investigate the suitability of gVLM, i.e. the sum of gVL-3′/4′-sulphate and gVL-3′/4′-*O*-glucuronide, as nutritional biomarkers of flavan-3-ol intake, and investigated whether or not it is suitable for the investigation of flavan-3-ol intake in large cohort studies.

## Methods

### Analytical method

gVL metabolites were analysed using UPLC-tandem MS. *De novo* synthesised authentic standards^22,23^ of the human gVL metabolites 5-(3′,4′-dihydroxyphenyl)-γ-valerolactone-3′-sulphate, 5-(3′,4′-dihydroxyphenyl)-γ-valerolactone-3′-*O*-glucuronide, as well as an isotope labelled internal standard, ^13^C_2_D_2_-5-(3′,4′-dihydroxyphenyl)-γ-valerolactone-3′-sulphate, were obtained from Analyticon (Analyticon Biotechnologies AG, Lichtenfels, Germany). Stock solutions were prepared in ethanol:water (70:30) and stored at −80 °C.

Flavan-3-ol-metabolite-free urine was obtained from human-volunteers on a low flavan-3-ol diet. This study was approved by the University of Reading Research Ethics Committee. All subjects gave their written informed consent to participate and all experiments were performed in accordance with relevant guidelines and regulations.

Spot urine samples (60 µL) and internal standard solutions (2.5 µM ^13^C_2_D_2_-5-(3′,4′-dihydroxyphenyl)-γ-valerolactone-3′-sulphate, 60 µL) were combined, filtered (Impact Protein Precipitation filter plate, Phenomenex, Macclesfield, UK) by centrifugation (500 × g) for 2 minutes at room temperature and stored at −20 °C until analysis. Sample preparation was automated for the analysis of EPIC Norfolk urine samples using a Hamilton Star robot (Hamilton, Bonaduz, Switzerland).

Samples were separated by liquid chromatography (Acquity, Waters, Elstree, UK) using a C18 column (Kinetex C18 200 × 2.1 mm, 1.7 µm, with 0.5 µm Krudcatcher, Phenomenex, Macclesfield, UK) and the conditions described in Table 1.

**Table 1:** HPLC-conditions for sample separation (flow rate 0.5 mL/min; column temperature 25 °C).

Time [min]	methanol:acetonitrile (10:90, v/v)	0.1% aqueous formic acid
Initial	5	95
2.00	5	95
3.50	15	85
6.00	20.4	79.6
6.10	95	5
7.00	95	5
7.10	5	95
8.00	5	95
Injection volume	7 µL (nominally – adjusted when required)	
Strong wash	40:30:30 Acetonitrile:Isopropanol:0.1% formic acid (aq)	
Weak wash	95:5 Water:Methanol	

Compounds were detected by electrospray ionisation tandem mass spectrometry (Applied Biosystems API 4000, Sciex, Warrington, UK) in negative ion mode using the parameters shown in Table 2. The spray voltage was −4500 V and the source temperature was 600 °C.

**Table 2:** LC-MS parameters for analytes and internal standards.

Analyte	Precursor ion [m/z]	Product ion [m/z]	Retention time [min]
5-(3′,4′-dihydroxyphenyl)-γ-valerolactone-3′-sulphate	287	207	3.80
5-(3′,4′-dihydroxyphenyl)-γ-valerolactone-3′-*O*-glucuronide	383	207	3.95
^13^C_2_D_2_-γ-valerolactone-3′-sulphate	291	211	3.78

Compounds were detected in negative ion mode with a dwell time of 20 ms.

Samples were quantified using calibration standards prepared in flavan-3-ol-metabolite free urine samples (standard concentrations [µM]: 0.1, 0.25, 10, 20, 30, 40, 50). 5-(3′,4′-dihydroxyphenyl)-γ-valerolactone-3′-*O*-glucuronide and 5-(3′,4′-dihydroxyphenyl)-γ-valerolactone-3′-sulphate were quantified using the peak area ratios of analyte and ^13^C_2_D_2_-5-(3′,4′-dihydroxyphenyl)-γ-valerolactone-3′-sulphate.

Each batch included two replicates of quality control samples with three different concentrations: low QC (0.3 µM), medium QC (25 µM) and high QC (38 µM). Usual acceptance criteria for each batch were that at least one QC at each concentration and four out of the six QCs were within 15% of the theoretical concentration.

The method was validated using flavan-3-ol-metabolite-free urine, spiked with the authentic standards 5-(3′,4′-dihydroxyphenyl)-γ-valerolactone-3′-*O*-glucuronide and 5-(3′,4′-dihydroxyphenyl)-γ-valerolactone-3′-sulphate to assess stability, specificity, matrix effects, precision and accuracy. The matrix effect was assessed by spiking flavan-3-ol-metabolite free urines from different sources with known amounts of analyte and internal standard and comparing their peak-area-ratio ratios. Method performance was assessed using data from quality control samples in 255 batches analysed.

### Human intervention studies

#### Test materials

Test materials consisted of fruit-flavoured non-dairy drinks that contained either specific flavan-3-ols (specificity study) or varying amounts of flavan-3-ols derived from cocoa (4 levels for the intake amount escalation study). Test materials were freshly prepared on each study day. All test materials were matched regarding their macro- and micro-nutrient content, including theobromine and caffeine, as well as in their orosensory and physicochemical characteristics. Composition of test materials is listed in Table 3. All test materials were supplied by Mars, Incorporated (McLean, VA).

**Table 3:** Composition of test materials.

Values (mg/70 kg BW)	Level 1	Level 2	Level 3	Level 4
Amount of CF	100.0	200.0	400.0	1000.0
Procyandins	81.7	163.3	326.7	816.7
(−)-Epicatechin	16.0	32.0	64.0	160.0
(+)-Catechin	0.4	0.9	1.8	4.4
(−)- Catechin	1.9	3.8	7.6	18.9
(+)-Epicatechin	0.0	0.0	0.0	0.0
Caffeine	18.1	18.4	19.1	21.1
Theobromine	97.6	97.3	96.9	95.6
Total calories	33.3	33.3	33.3	33.3
Total Carbohydrates	7.8	7.8	7.8	7.8
Sodium	6.7	6.7	6.7	6.7

#### Study population

Healthy male adults between 25 and 60 years of age (specificity study) and between 25 and 40 years of age (intake amount escalation study) were recruited by public advertisement in the city of Davis and surrounding areas (California, USA). Exclusion criteria included a body mass index (BMI) higher than 30 kg/m^2^, blood pressure (BP) higher than 140/90 mmHg, allergies to peanut or cocoa, avoidance of caffeinated food products and beverages, a history of CVD, stroke, renal, hepatic, or thyroid disease, gastrointestinal tract disorders, previous gastrointestinal surgery (except appendectomy), the current intake of herbal-, plant- or botanical-containing dietary supplements, persons following vegan/vegetarian diet, and those adhering to an uncommon diet or a weight loss program. To determine eligibility, participants were asked to complete health- and lifestyle questionnaires, have their height, weight, and in-office BP determined, and to provide a blood sample for complete blood count (CBC), liver panel, lipid panel and metabolic panel assessments. Enrolled participants commenced the study protocol between 1–3 weeks after eligibility was determined. While participating in the study, volunteers were asked to maintain their typical daily activities and diet throughout the study. To control for potential dietary flavan-3-ol intake, volunteers were asked to follow a defined low-flavan-3-ol diet on the day prior to and during each study day. All volunteers were instructed on how to follow a low-flavan-3-ol diet, receiving foods containing low or negligible amounts of flavanols including the dinner for the night previous to the study day. Additionally, volunteers were asked to restrain from consuming alcohol, coffee, or any other caffeine-containing beverages for one day prior, and during, the study days. Volunteers were required to fast overnight (12 h water, *ad libitum*) before each study day.

#### Specificity study

In order to investigate the specificity of gVLM, the sum of gVL-3′/4′-sulphate and gVL-3′/4′-*O*-glucuronide, as nutritional biomarker of flavan-3-ol intake, we performed a dietary intervention study with different possible precursors of gVL. This study was randomized, double-masked and followed an 8x -crossover desig n, in which volunteers were asked to consume test materials containing specific flavan-3-ols along with toast with cream cheese. The flavan-3-ols tested were: i) (−)-epicatechin ii) (−)-epigallocatechin, iii) (−)-epicatechin-3-*O*-gallate, iv) (−)-epigallocatechin-3-*O*-gallate (all compounds isolated from green tea, purity >99% and food grade), v) a 1:1:1: mixture of theaflavin-3-*O*-gallate, theaflavin-3′-*O*-gallate and theaflavin-3,3′-*O*-digallate, vi) thearubigins (all isolated from black tea, purity >95% and food grade) and vii) procyanidin B-2 ((−)-epicatechin-(4α4β)-(−)-epicatechin; isolated from cocoa, purity 91%). One additional visit included the consumption of the test material without any flavan-3-ol added (control day). The amount of (−)-epicatechin consumed per volunteer was 34.8 mg or 120 µmol, which is close to the mean intake amount of (−)-epicatechin consumed in the UK^24^. The amount of the rest of the flavan-3-ols was equimolar to the amount of (−)-epicatechin ingested, except for thearubigins, which were given in equal mg amounts to the theaflavin mix.

After intake, urine was collected over 24 h in two collection periods (from 0 h to 6 h post intake and 6 h to 24 h post intake), using a fresh container for each collection period with 20 ml of 2 M sodium acetate (pH = 4.5) and 2 mL of 0.5% (w/v) thymol in isopropanol as preservatives. Volunteers returned containers upon completion of sample collection, and urine was stored at −80 °C until analysis. The minimum sample size required to detect a minimum change of 6 µmol of gVLM in 24 h-urine (equivalent to a 5% of the amount of flavanols ingested) was determined by pair-t test and imputing a standard deviation of 4 µmol. Volunteers were randomized to receive the 8 different test materials using computer-generated lists of random numbers via the randomly permuted block method. The allocation list was generated by a researcher not involved with the recruitment and allocation of participants. We assessed 17 volunteers for eligibility, 12 volunteers were enrolled in the study, and 8 volunteers completed all 8 interventions with the rest of the enrolled volunteers completing <4 interventions. Participants, study nurses, and researchers assessing outcomes as well as researchers involved with the statistical analysis of data were masked to the specific nature of intervention. The initial recruitment started in August 2016, and the study was conducted from August 2016 to January 2017. This study protocol was approved by the Institutional Review Board of UC Davis. All subjects gave their written informed consent to participate and all experiments were performed in accordance with relevant guidelines and regulations. This study was registered as NCT03194620.

#### Intake amount escalation study

In order to investigate the association between flavan-3-ol intake and gVLM excretion, we conducted a dietary intervention study where participants consumed different amounts of flavan-3-ol. This study was randomized, double-masked and followed a 4 × -crossover design, in which volunteers consumed test materials containing four different amounts of flavan-3-ols from cocoa. These flavan-3-ols include (−)-epicatechin, (+)- and (−)-catechin and their oligomers, the procyanidins, with a degree of polymerization from 2 to 10 in a profile analogous to that naturally found in the seeds of *Theobroma cacao L*. On four separate occasions, each volunteer consumed four different amounts of flavan-3-ols that ranged from 95 mg, including 15 mg of (−)-epicatechin to 1424 mg, including 227 mg of (−)-epicatechin, which are amounts well within the range of intakes determined in the UK^24^. After intake, urine was collected over 24 h in 4 collection periods (from 0 h to 4 h post intake, 4 h to 8 h post intake, 8 h to 12 h post intake and 12 h to 24 h post intake), using a fresh container for each collection period with 20 ml of 2 M sodium acetate (pH = 4.5) and 2 mL of 0.5% (w/v) thymol in isopropanol as preservatives. Volunteers returned the container upon completion of sample collection, and urine was stored at −80 °C until analysis. The minimum sample size required to detect changes of 20 µmol of gVLM in 24 h-urine after the intake of each test drink was determined by ANOVA and imputing a standard deviation of 14 µmol. Volunteers were randomized to receive the four different test materials using computer-generated lists of random numbers via the randomly permuted block method. The allocation list was generated by a researcher not involved with the recruitment and allocation of participants. We assessed 20 volunteers for eligibility, and 14 volunteers were finally enrolled and completed the study. Participants, study nurses, and researchers assessing outcomes as well as researchers involved with the statistical analysis of data were masked to the intervention. The initial recruitment started in April 2013, and the study was conducted from April to May 2013. This study protocol was approved by the Institutional Review Board of UC Davis. All subjects gave their written informed consent to participate and all experiments were performed in accordance with relevant guidelines and regulations. This study was registered as NCT03201822.

#### Sample analysis

Urine samples from human intervention studies (50 µL) were diluted with 100 µL of 100 µM 3-methyl hippuric acid (internal standard) and analyzed by UPLC-MS within 24 h. Samples were separated on a Waters Acquity UPLC (Waters, Milford, MA, USA) with an autosampler cooled at 5 °C. The injection volume was 37.5 µL. Chromatography was carried out at 25 °C using a 100 × 2.1 mm Kinetex C18 1.7 µm, reversed phase column with a krudcatcher (Phenomenex). The mobile phase, pumped at a flow rate of 0.5 mL/min, was (A) 5 mM ammonium formate (pH 2.9); and (B) acetonitrile/methanol (90:10, v/v). The gradient started with 5% B from 0–2 min, then ramped to 17.5% from 2–3.5 min, and then to 22.5% from 3.5–6 min. Detection was achieved with a Waters Quattro Micromass spectrometer fitted with an electrospray interface (ESI; Waters). MS analysis was carried out in negative ionization mode by multiple reaction monitoring (MRM). Detection of gVL-3′-sulphate and -3′-*O*-glucuornide utilized transitions from 287 m/z to 207 m/z and 383 m/z to 207 m/z, respectively, and both with a dwell time of 0.02 s, a cone voltage of 32 V, and a collision energy of 20 V. Data processing was performed using Masslynx software (Waters). MS conditions were set by tuning with the corresponding compound. Source and desolvation temperature were set at 150 °C and 500 °C, respectively, while desolvation and cone gas were set at 900 L/h and 30 L/h, respectively. In both ionization modes, capillary, cone, and lens voltage were set at 4 kV, 30 V, and 1 V, respectively. The identification and quantification of the compounds were based on co-elution with authentic standards. The precision and accuracy of the method was better than 5% and 20%, respectively.

### EPIC Norfolk

We have investigated the feasibility of using gVLM as a nutritional biomarker in a large observational cohort by applying the method developed in Section 2.1 to 5000 random spot urine samples of the EPIC Norfolk cohort^25^. Participants of this study (n = 25,639), healthy men and women between 40 and 75 years living in and around Norwich, UK, were recruited between 1993 and 1997. Spot urine samples were collected during the baseline health examination and stored at −20 °C prior to analysis. The study was approved by the Norwich District Health Authority Ethics Committee, and all participants gave signed informed consent.

### Statistical analyses

Associations between intake and biomarker were assessed with linear regression models using R 3.4.2^26^ with the boot package^27^. Adjusted R^2^ estimates were obtained by bootstrapping (n = 10,000).

### Data availability

The datasets generated during and/or analysed during the current study are available from the corresponding author on reasonable request.

## Results

### Analytical method validation and performance

**Figure 1:**
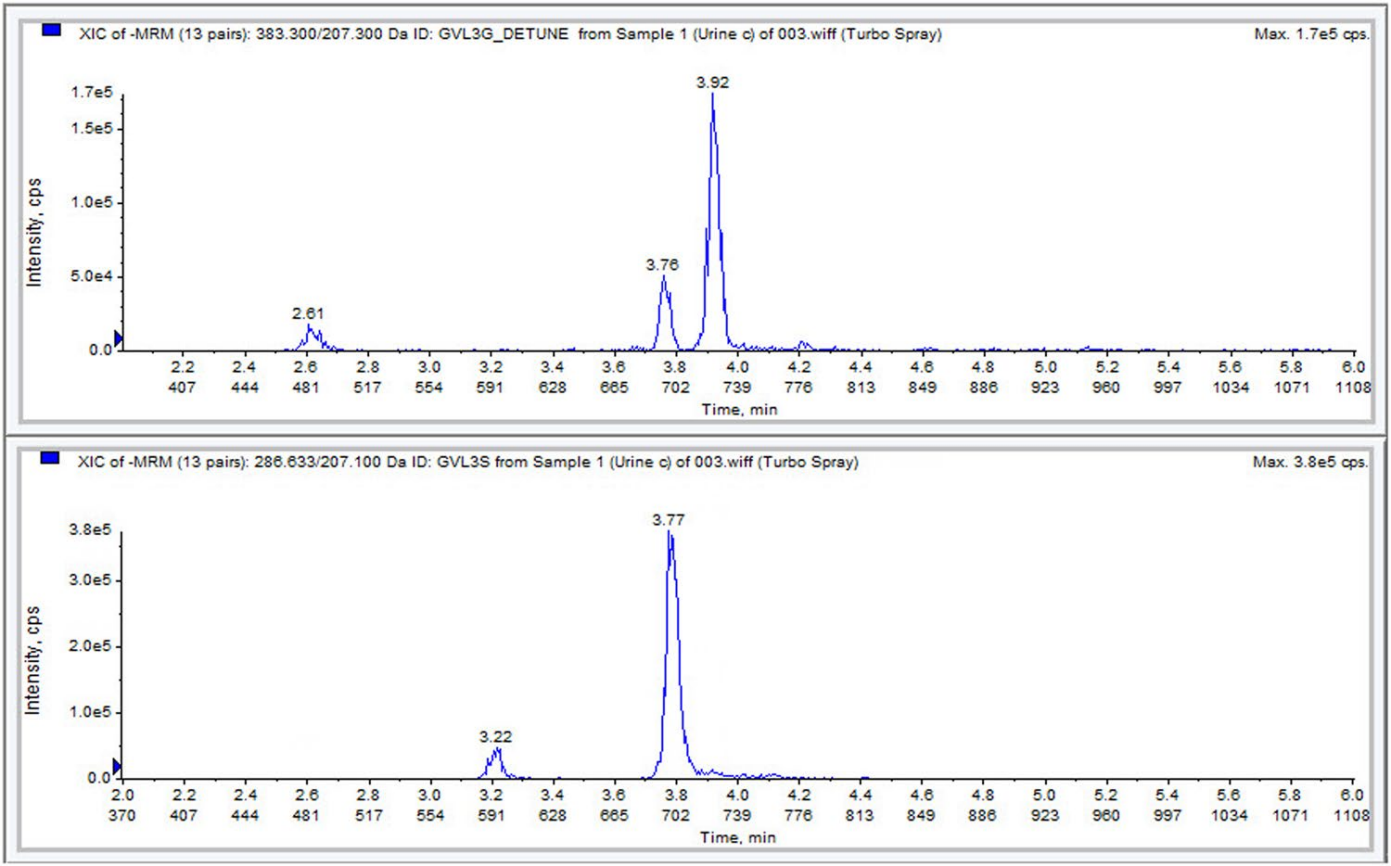
Typical chromatogram of EPIC Norfolk spot urine sample, showing γ-valerolactone-3′/4′-*O*-glucuronide (top, 3.9 minutes) and γ-valerolactone-3′-sulphate (bottom, 3.8 minutes) in urine.

The application of a biomarker for nutrient intake assessments requires a robust, validated analytical method, which is ideally suitable for automation and high-throughput analysis. The method developed here can be highly automated and adopted to a robotic sample preparation and analysis platform, which allows for the large-scale analysis of gVLM within an 8 minute run time. Figure 1 shows a typical chromatogram of gVL-3′/4′-sulphate and gVL-3′/4′-*O*-glucuornide. The precision and accuracy of the method was well within the usual GLP criteria of 15%, and long-term performance monitoring of 255 batches over more than 100 days showed a typical accuracy of better than 5% and a precision better than 10% (Table 4). The estimated limit of detection (signal-to-noise ratio better than 5-to-1) was at least 0.1 µM. There was a strong matrix effect, which was adequately compensated for by the isotope-labelled internal standard. All compounds were tested for their stability in urine under different conditions, including at high temperature (six hours at 35 °C, 24 h hours at room temperature) and following four freeze-thaw cycles (−80 °C), and the extracted samples were tested for 72 h hours at 5 °C and seven days at −20 °C. All compounds were stable under these conditions and showed no indication of deterioration.

**Table 4:** Long-time precision (%CV) and accuracy (%RE, difference of mean calculated concentration and nominal concentration, standardised by nominal concentration) based on 288 analysed batches.

	Mean (SD) [µM]	%CV	%RE	Mean (SD) [µM]	%CV	%RE	Mean (SD) [µM]	%CV	%RE
	Low QC (0.30 µM)			Medium QC (25 µM)			High QC (38 µM)		
5-(4′-hydroxyphenyl)-γ-valerolactone-3′-sulphate	0.29 (0.02)	8.2	−4.6	24.9 (1.1)	4.4	−0.3	37.9 (1.5)	3.9	−0.4
5-(4′-hydroxyphenyl)-γ-valerolactone-3′-*O*-glucuronide	0.30 (0.04)	11.9	−1.1	25.4 (2.2)	8.7	1.9	37.9 (2.9)	7.7	−0.3

**Figure 2:**
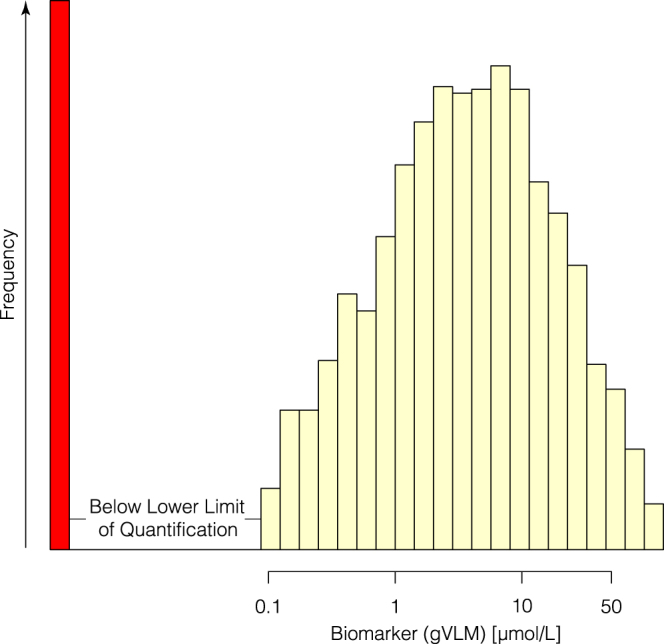
Distribution of gVLM (sum of γ-valerolactone-3′/4′-sulfate and γ-valerolactone-3′/4′-*O*-glucuronide) concentrations in 5000 random samples of EPIC Norfolk. The red bar indicates samples with concentration below the lower limit of quantification (0.1 µmol/L).

In 5000 random spot urine samples of EPIC Norfolk, median (IQR) concentration of the candidate biomarker (i.e. the sum of gVL-3′/4′-sulphate and gVL-3′/4′-*O*-glucuornide) was 3 (0.8–10.1) µmol/L, and in 421 (8%) samples the biomarker concentration was below the lower limit of quantification (0.1 µmol/L). The distribution of biomarker concentrations (Fig. 2) was skewed to the right and virtually log-normal.

### Specificity of 5-(3′,4′-dihydroxyphenyl)-[gamma]-valerolactone as candidate biomarker of flavan-3-ol intake

**Figure 3:**
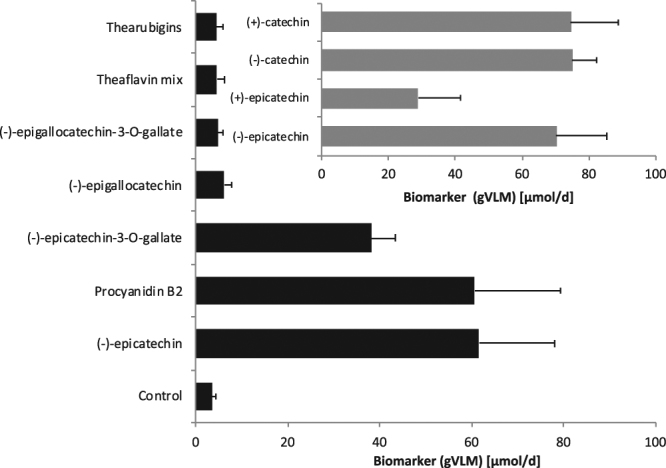
Urinary excretion of flavan-3-ol biomarker (gVLM, sum of γ-valerolactone-3′/4′-sulphate and O–glucuronide) following the consumption of different flavan-3-ols (error bars show standard error; n = 12–8). *indicate statistically significant differences (p < 0.05; repeated measures ANOVA).

**Figure 4:**
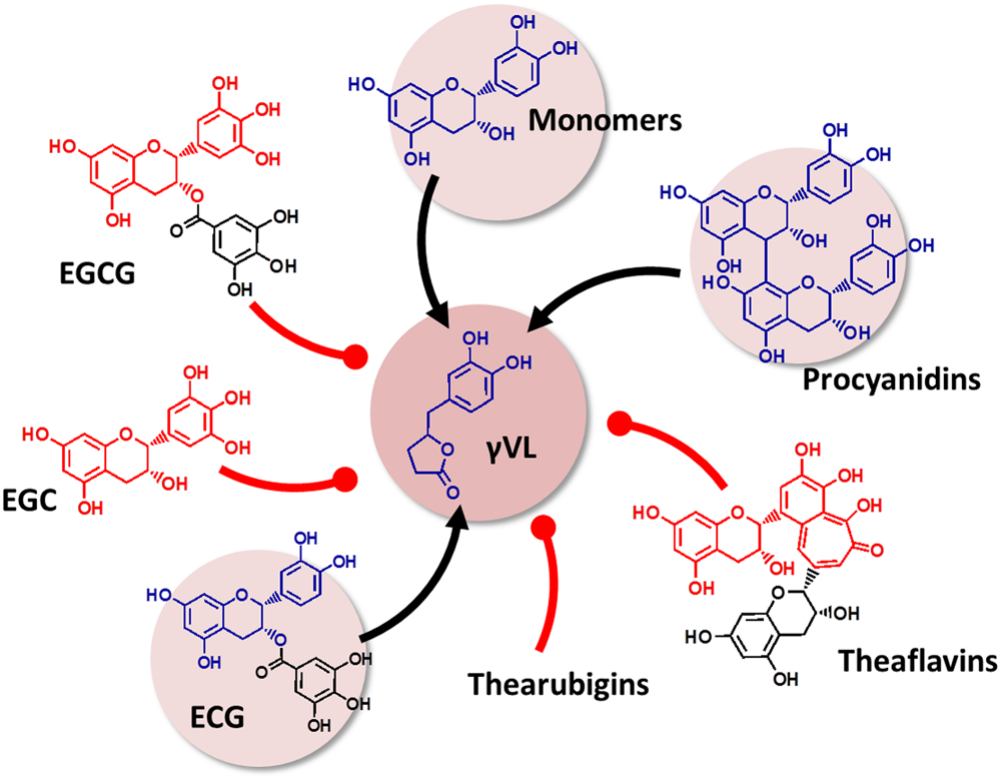
Flavan-3-ol precursors of the microbial metabolite 5-(3′/4′-dihydroxyphenyl)-γ-valerolactone (gVL). Only compounds with intact (epi)catechin moiety result in the formation of γVL by the intestinal microbiome. ECG, (−)-epicatechin-3-*O*-gallate; EGCG, (−)-epigallocatechin-3-*O*-gallate; EGC, (−)-epigallocatechin.

In humans, gVL is formed by the colonic microbiome from different flavan-3-ol precursors^18,19^. However, flavan-3-ols are a complex group of compounds and there was insufficient information about the type of flavan-3-ol that results in the formation of gVL. We have therefore investigated the formation of gVL in humans following the consumption of different monomeric and oligomeric and steroisomeric flavan-3-ols. This study showed that the oral intake of (−)-epicatechin, (−)-epicatechin-3-*O*-gallate and procyanidin B-2 resulted in the formation and subsequent presence in urine of both, gVL-3′/4′-sulphate and gVL-3′/4′-*O*-glucuronide. However, the consumption of theaflavins, thearubigins, (−)-epigallocatechin and (−)-epigallocatechin-3-*O*-gallate, did not result in the formation of gVL, thus did not contribute to the presence of any of the gVLM in urine. Notably, the intake of equimolar amounts of procyanidin B-2 and (−)-epicatechin-3-O-gallate resulted in similar amounts of gVL-3′/4′-sulphate and −3′/4′-*O*-glucuronide as (−)-epicatechin (Fig. 3). To further characterize the specificity of gVLM, we have also re-analysed a subset of urine samples collected after the intake of different (epi)catechin stereoisomers in humans^28^, demonstrating that all (epi)catechin stereoisomers also contribute to the presence of gVL-3′/4′-sulphate and -3′/4′-*O*-glucuronide in urine (Fig. 3, insert). In summary, only flavan-3-ols with an intact (epi)catechin moiety can contribute to the formation of gVL and subsequently urinary gVLM (Fig. 4).

### Association between flavan-3-ol intake and gVLM

**Figure 5:**
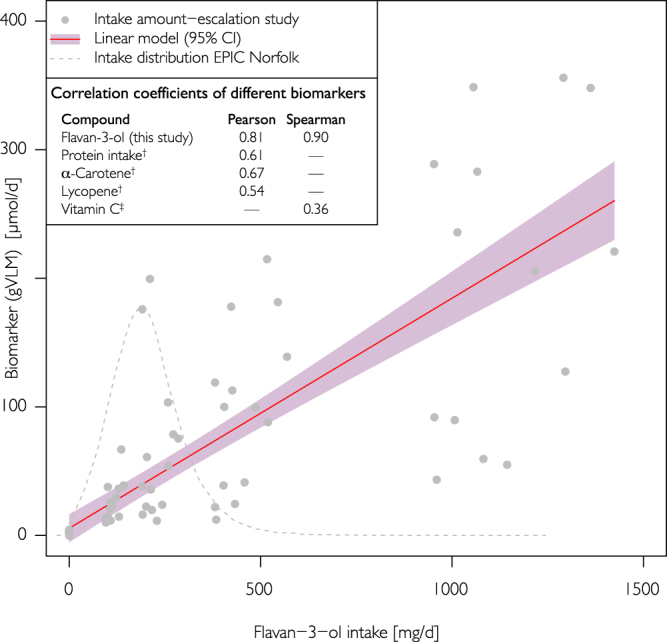
Association between flavan-3-ol intake and gVLM (sum of 5-(3′/4′-dihydroxyphenyl)-γ-valerolactone-3′/4′-sulphate and O–glucuronide metabolites). Results from the intake-amount escalation study and regression analysis (R^2^ = 0.66; 95% CI 0.49; 0.80). The intake amount used is comparable with the estimated habitual intake in EPIC Norfolk^6^. For comparison, Pearson and Spearman correlation coefficients for other biomarkers are shown (^†^^44^; ^‡^^55^).

A strong and consistent association between flavan-3-ol intake and the gVLM is crucial for their use as biomarker of intake. We have therefore investigated this association using daily intakes in humans of up to 1400 mg/d of flavan-3-ols, which covers the entire range of flavan-3-ol intake observed in EPIC Norfolk (0–1200 mg/d^6^). The results of our intake amount-escalation study show a strong and consistent association between flavan-3-ol intake and 24 h urinary excretion of gVLM (R^2^ = 0.66; 95% CI 0.49; 0.80; Spearman’s rank correlation: ρ = 0.90; Pearson’s r = 0.81; Fig. 5). These associations were attenuated, but remained significant, when excluding flavan-3-ol amounts higher than the top decile of intake in EPIC Norfolk (325 mg/d), with R^2^ = 0.50 (95% CI 0.34; 0.70), a Spearman’s rank correlation coefficient of ρ = 0.82 and a Pearson’s r of r = 0.70. A similar attenuation was observed when excluding data for a flavan-3-ol intake of 0 mg/d (R^2^ = 0.49 (95% CI 0.25; 0.69), Spearman’s rank correlation coefficient ρ = 0.74; Pearson’s r of r = 0.70).

## Discussion

In this study, we have assessed the suitability of the 5-(3′,4′-dihydroxyphenyl)-γ-valerolactone (gVL) phase II metabolites, gVL-3′/4′-*O*-glucuronide and gVL-3′/4′-sulphate (gVLM), as nutritional biomarker of flavan-3-ol intake, using the criteria suggested by IARC^10^ and the Institute of Medicine (IOM)^21^. In the course of this assessment, we (i) developed a robust, precise and accurate method to analyse at scale gVLM in urine samples, (ii) established which flavan-3-ols lead to the formation of gVLM in humans, and (iii) showed a consistent relationship between flavan-3-ol intake and urinary concentrations of gVLM. In the following section, we discuss whether or not gVLM meets each of these criteria.

### Measurement of gVLM as nutritional biomarker

The application of a nutritional biomarker requires a robust, precise and accurate bioanalytical method to determine the biomarker in the respective biospecimen. Here, we use phase II metabolites of gVL, and their accurate quantification requires the availability of authentic standards as all other methods introduce bias. The most commonly used approach to date, the deconjugation with sulphatases and ß-glucuronidase enzymes, followed by the analysis of the aglycon, relies on the complete and consistent hydrolysis of phase II metabolites. However, we and others have shown previously that this cannot be fully and consistently achieved for metabolites of flavan-3-ols^29,30^ and that the degree of hydrolysis is affected by reaction conditions^31^. An alternative method not relying on hydrolysis is to identify metabolites by their characteristic tandem-MS fingerprint and to quantify them as aglycone. However, this also introduces large instrument- and method-dependent variability, which renders the results not suitable for biomarker analysis. Authentic standards thus remain the “gold standard” for the identification and quantification of these compounds^32^, and the development of this method was therefore made possible by the availability of authentic standards^22,23^.

Isotope-labelled standards are often used in tandem mass spectrometry for quantification, especially as they can compensate for matrix effects^33^. However, this can be affected by slight differences in retention time between labelled and unlabelled standard^34^ due to the isotope effect^35^, resulting in an uncompensated or partially uncompensated matrix effect. For gVLM, the matrix effects were adequately compensated and precision and accuracy for both compounds studied here were well within the accepted range with an error of less than 15%.

The validation of the method developed here was shown to be robust, accurate and precise, and has been validated with reference to generally accepted standards^36^. Furthermore, with a run time of 8-minutes and an easily automatable sample preparation, we show that this method is suitable for robotic automation and therefore for the high-throughput analysis of samples for the application of this method to large-scale studies. The application to a random subset of samples collected from EPIC Norfolk shows that the validation range is sufficient to measure the biomarker in the majority of participants.

### gVLM are specific for flavan-3-ols

While the relationship between nutritional biomarker and intake is often assessed^11,12^, there is a paucity of data on their specificity. Indeed, it is often tacitly assumed that structurally-related metabolites, such as, for example glucuronidated or sulphated forms, are sufficiently specific for individual compounds^37,38^, but this assumption is rarely proven out. Investigating specificity is especially important when developing a biomarker for a structurally diverse group of compounds, such as flavan-3-ols.

Flavan-3-ols are extensively metabolised by the colonic microbiome^18,39^, but many of the metabolites, such as hippuric acid and phenylacetic acids are also derived from many other precursors^40^, and therefore not suitable to assess their intake. Urinary gVLM in humans has been found only after the intake of flavan-3-ols, and not following the consumption of other flavonoids and polyphenols^17–19^. However, flavan-3-ols encompass a complex group of compounds (Fig. 3), and little was known about the contribution of specific flavan-3-ols to the presence of gVLM in urine. The results obtained here showed that human consumption of (−)-epicatechin, epicatechin-3-*O*-gallate and procyanidin B-2 resulted in the formation of gVL, whereas theaflavins, thearubigins, (−)-epigallocatechin and (−)-epigallocatechin-3-*O*-gallate did not. We also showed that the intake of (−)-epicatechin stereoisomers contributes to the presence of gVL, confirming previous reports^17,18^. The finding that epicatechin-3-*O*-galllate intake gives raise to gVLM in urine implies that epicatechin-3-O-galllate is hydrolysed after intake, and that the resulting epicatechin is subsequently converted into gVL by the colonic microbiome. Thus, gallic acid esters of other (−)-epicatechin stereoisomers, like catechin-3-*O*-galllate, are likely to also undergo this transformation. Similarly, other dimeric procyanidins different from procyanidin B-2, like procyanidin B1 [(−)-epicatechin-(4αß)-(+)-catechin], should be expected to contribute to urinary gVLM.

The data available today does not allow for a direct assessment of whether or not procyanidins with a higher degree of polymerisation (DP > 2) contribute to urinary gVLM. However, our data show that only one of the (−)-epicatechin units in procyanidin B2 contributed to the presence of gVLM in urine, and it is likely that this will also be applicable to procyanidins with a higher degree of polymerisation, i.e. that if the terminal unit is a (epi)catechin moiety it gives rise to the formation of gVL. Taken together, our results show that urinary gVLM are specific for the intake of (−)-epicatechin and its stereoisomers, their 3-O-gallate esters and procyanidins.

### Relationship between dietary intake and gVLM

The intended use of a nutritional biomarker is to provide data on dietary intake, and a consistent relationship between dietary intake of a given compound and its biomarker of intake is therefore crucial. This requires an accurate and precise estimate of actual intake, but many studies rely on intake assessments derived from self-reporting methods and food-composition data as reference to validate nutritional biomarkers^41^. This is done despite the known limitations of data derived from self-reporting and food-composition methods^7,8^, especially with regard to a high variability in food content^41,42^. An investigation of the intake-biomarker relationship should therefore either use duplicate diets with validated analytical methods to assess intake, or use defined dietary interventions. In this study, we have used dietary interventions for which detailed compositional data were available (Table 3), and our data show a strong and consistent association between flavan-3-ol intake and urinary gVLM levels. The R^2^ value of 0.66 and correlation coefficient of 0.90 are comparable or better than that of many other well-accepted nutritional biomarkers^43,44^, and only moderately attenuated when excluding upper extremes of intake. Importantly, this strong association was possible even in the context of expected inter-individual differences in the production of gVL^45^. In this manner, gVLM differs from other gut microbiome-derived flavonoid metabolites, like equol, which shows large inter-individual differences, even capable of distinguishing populations as equol producers and non-producers^46^. In this context, and limited by the rather small sample cohort studies, we have previously reported that gVL metabolites show inter-individual differences of 60% in humans^47^. This inter-individual variability of gVL metabolites is more comparable to that reported for other gut microbiome metabolites, like enterolatone and enterodiol^48^, which are well recognized biomarkers of lignan intake^49^.

### Suitability of gVLM as nutritional biomarker of flavan-3-ol intake

Taken together our results show that gVLM fulfils the criteria set to evaluate candidate nutritional biomarkers, and that gVLM qualifies as a surrogate biomarker^13^ of flavan-3-ol intake. It therefore enables the ranking of participants according to their flavan-3-ol intake and thus enables investigations into associations with disease risk biomarkers^10–12^ or morbidity/mortality endpoints. While the validation of gVLM has been conducted with 24 h urine samples, surrogate biomarkers can also rely on the use of spot urines. In this context, the plasma half-life of gVLM of approximately 6 h, (6.3 ± 1.7 h for gVL-3′-sulphate and 3.1 ± 0.6 h for gVL-3′-*O*-glucuronide)^17^, is sufficient to reach steady-state conditions with regular intake, which is the case for most dietary sources of flavan-3-ols, such as tea and various fruit. Therefore, it can be expected that the urinary excretion of gVLM is sufficiently constant to allow the use of creatinine-adjusted^50^ biomarker concentration in spot urine samples. Indeed, data for enterolignans, colonic metabolites with similar half-lives (enterodiol, 4.4 ± 1.3 h; enterolactone 13 ± 5.6 h^51^) to that of gVLM, show very high correlation between spot and 24 h urine concentrations^52^. Frequent high flavan-3-ol intake will therefore result in consistently higher gVLM concentrations in spot urine than low or episodic intake.

The data presented here, in combination with pharmacokinetic data published previously^17^, therefore show that creatinine-adjusted gVLM concentration in spot urine samples can be used as nutritional biomarker of flavan-3-ol intake.

## Conclusion

In this study, we have evaluated the suitability of the microbiome-derived flavan-3-ol metabolite, 5-(3′,4′-dihydroxyphenyl)-γ-valerolactone (gVL), as nutritional biomarker for the assessment of flavan-3-ol intake in large-scale epidemiological studies. The development of this biomarker has been made possible by the availability of authentic standards to allow the quantification of the Phase II metabolites gVL-3′/4′- sulphate and gVL-3′/4′-*O*-glucuronide in urine. The method developed is suitable for automation and thus high-throughput analysis, and we have applied it to 5000 random samples of the EPIC Norfolk cohort. The results show that the biomarker is found in the majority of participants with a wide range of concentrations and a high variability, allowing for the ranking of participants according to flavanol-3-ol intake.

We could show that this nutritional biomarker is specific for a number of specific flavan-3-ols present in tea, fruits, wine and cocoa-derived products, which represent the main sources of flavan-3-ols in the diet^53,54^. Overall, the biomarker established here allows for a significantly improved approach in the investigation of the associations between flavan-3-ol intake and health outcomes in large-scale observational studies, thereby providing critical data in the context of developing potential dietary recommendations.
